# Plants as Sources of Anti-Inflammatory Agents

**DOI:** 10.3390/molecules25163726

**Published:** 2020-08-15

**Authors:** Clara dos Reis Nunes, Mariana Barreto Arantes, Silvia Menezes de Faria Pereira, Larissa Leandro da Cruz, Michel de Souza Passos, Luana Pereira de Moraes, Ivo José Curcino Vieira, Daniela Barros de Oliveira

**Affiliations:** 1 Laboratório de Tecnologia de Alimentos, Centro de Ciências e Tecnologias Agropecuárias, Universidade Estadual do Norte Fluminense Darcy Ribeiro, Campos dos Goytacazes, RJ 28013-602, Brazil; clara_biol@yahoo.com.br (C.d.R.N.); mariana.arant@yahoo.com.br (M.B.A.); silvia@uenf.br (S.M.d.F.P.); larissa.leandrocruz@gmail.com (L.L.d.C.); luana@uenf.br (L.P.d.M.); 2 Laboratório de Ciências Químicas, Centro de Ciências e Tecnologia, UniversidadeEstadual do Norte Fluminense Darcy Ribeiro, Campos dos Goytacazes, RJ 28013-602, Brazil; michelpassos19@gmail.com (M.d.S.P.); curcino@uenf.br (I.J.C.V.)

**Keywords:** drugs, inflammation, bioactive compounds

## Abstract

Plants represent the main source of molecules for the development of new drugs, which intensifies the interest of transnational industries in searching for substances obtained from plant sources, especially since the vast majority of species have not yet been studied chemically or biologically, particularly concerning anti-inflammatory action. Anti-inflammatory drugs can interfere in the pathophysiological process of inflammation, to minimize tissue damage and provide greater comfort to the patient. Therefore, it is important to note that due to the existence of a large number of species available for research, the successful development of new naturally occurring anti-inflammatory drugs depends mainly on a multidisciplinary effort to find new molecules. Although many review articles have been published in this regard, the majority presented the subject from a limited regional perspective. Thus, the current article presents highlights from the published literature on plants as sources of anti-inflammatory agents.

## 1. Introduction

The magnitude of global plant diversity is estimated at more than 500,000 species [1,2], and the variety and complexity of plant metabolites represent a challenge when considering exploration of the chemical repertoire offered. From this point of view, the Plant Kingdom has been pragmatic, especially when these molecules are reported as substances with the high medicinal potential to treat diseases that affect living beings [3].

Medicinal plants continue to be an interesting source of natural products for treating various health conditions. It is estimated that more than 150,000 plant species have been studied, many of which contain valuable therapeutic agents, and the applications of novel compounds from plants for pharmaceutical purposes have been gradually increasing in recent years [4,5].

Plants have played an important role in human health care since ancient times. In an adaptation against attacking pathogen and environmental stress, plants produce several substances that exert biological activities. These small organic molecules come from secondary metabolism and have several biological activities. Among the diverse functions, anti-inflammatory actions are highlighted [6,7].

It is known that inflammation is an evolutionarily conserved process of protection and a critical survival mechanism [8]. It is composed of complex sequential changes in the tissue to eliminate the initial cause of the cell injury, which may have been caused by infectious agents or substances from their metabolism (microorganisms and toxins), as well as by physical agents (radiation, burn, and trauma), or chemicals (caustic substances) [9,10]. The signs of inflammation are local redness, swelling, pain, heat, and loss of function [7].

In general, this complex biological response leads to the restoration of homeostasis. However, in cases of prolonged release of inflammatory mediators and the activation of harmful signal-transduction pathways, the inflammatory process persists, and a mild but chronic proinflammatory state may arise [8]. A low-grade inflammatory state is correlated with various disorders and chronic health conditions, such as obesity, diabetes, cancer, and cardiovascular diseases, among others [11,12,13,14,15,16,17,18].

Therefore, the discovery of a new generation of therapeutic agents to use in the resolution of inflammation is desirable. The treatment of inflammation involves some mechanisms that can be used as therapeutic targets [8]. Due to the production of secondary metabolites with clinically curative effects, medicinal plants play an important role in the development of new and potent drugs [19,20].

Another motivating scientific investigation related to drugs and medicines made from plants is their interaction with gut microbiota. Certain gut bacteria intensively metabolize drugs rich in the low-molecular-mass products of secondary metabolisms, such as tannins and anthocyanins. Metabolites derived from bacterial metabolization are small, bioavailable, and potentially bioactive metabolites. They also have potential modulatory effects on the gut microbiome, which is interesting to prevent metabolic disorders [21].

## 2. Anti-Inflammatory Drugs

Anti-inflammatory drugs can interfere in the pathophysiology of inflammation, seeking to minimize tissue damage and provide greater patient comfort. The major classes of anti-inflammatory agents are the glucocorticoids and non steroidal anti-inflammatory drugs (NSAIDs). Fundamentally these differ in their mode of action. In short, glucocorticoids act by inhibiting prostaglandins and proteins involved in inflammatory processes, such as corticosteroids, which among other indications are used in treatment for asthma and autoimmune inflammatory response. Non-steroidal drugs, on the other hand, have an inhibitory action through the enzyme cyclooxygenase and are indicated for moderate and mild pain and body temperature control. An example of a non-steroidal drug is acetylsalicylic acid [22].

NSAIDs are the most commonly used drugs worldwide [7], utilized to treat acute and chronic pain resulting from an inflammatory process [22]. NSAIDs encompass a range of agents and, in general, all their effects are related to the inhibition of COX action in the production of prostaglandins and thromboxanes [23,24,25,26].

The main mechanism of action of NSAIDs is the inhibition of COX, both central and peripheral, interfering in the conversion of arachidonic acid to prostaglandins E2, prostacyclins, and thromboxanes. Enzymes related to the action of NSAIDs can be divided into COX-1 and COX-2, acting in different regions. COX-1 appears in most cells, even fetal and amniotic fluid, and participates in physiological effects, such as regulatory and protective effects. On the other hand, COX-2 is activated by inflammation and proinflammatory cytokines [27,28].

There are several ways to classify NSAIDs; according to COX-2 inhibitory potency over COX-1, concentration to achieve clinical effects, among others. NSAIDs can be classified into non-selective NSAIDs (ketoprofen, aspirin, naproxen, flunixin, meglumine, and others), preferential COX-2 inhibitors (meloxican, etodolac, nimesulide), and highly selective COX-2 inhibitors (coxib). Most of the side effects are related to the inhibition of COX-1 due to its performance in several systems related to cell cleansing. Besides, NSAIDs can also be classified according to their chemical structure (Table 1).

Structurally, COX-2 selective drugs contain sulfonamide groups or sulfones, responsible for the selectivity of the enzyme and do not have a carboxylic group and, therefore, they can selectively target the COX-2 enzyme. They have little water solubility, which hinders parenteral administration [30].

Acetylsalicylic acid (ASA) is one of the most widely used drugs in the world. It is used as an analgesic, antipyretic, and anti-inflammatory [31,32]. This drug also has antiplatelet or anticoagulant effects and is used to prevent heart attacks, strokes, and blood clots [33]. However, its use can also lead to exacerbated respiratory tract disease and cancer [32,34].

Historically, non-steroidal anti-inflammatory drugs, such as acetylsalicylic acid (Aspirin^®^), indomethacin, ibuprofen, and piroxicam have been used clinically for the treatment of inflammation due to their suppression of the effects of COX activity [24].

However, these traditional NSAIDs act in a non-selective manner inhibiting both forms of COX and have also demonstrated side effects. Specific modalities of anti-inflammatory effects and side effects are associated with the existence of two COX isoforms [35].

Inhibitory actions of aspirin or other non-steroidal anti-inflammatory drugs against COX-1 may present crucial problems in pharmacotherapy. Some anti-inflammatory drugs that act only to inhibit COX-1 are ibuprofen, naproxen, fenoprofen, ketoprofen, flurbiprofen, and oxaprozin (derivatives of propionic acid); indomethacin, sulindac, and etodolac (indoleacetic acids); piroxicam (derived from oxyanas); mefenamic acid and meclofenamate (phanamates); and diclofenac [29].

Thus, the scientific community has focused its efforts on the search for selective COX-2 inhibitors, with lower adverse side effects, since highly selective COX-2 inhibitors are required for the treatment of inflammatory diseases [35]. The first selective COX-2 inhibitor was celecoxib (Celebrex^®^), followed by rofecoxib (Vioxx^®^). In a short time, the coxibs (celecoxib and rofecoxib) have achieved wide dissemination [36]. Other drugs are also more selective for COX-2 than for COX-1, such as nimesulide and etodolac [37].

Despite initial success, shortly after the launch of selective COX-2 inhibitors, adverse cardiovascular and renal effects have been reported and, in high-doses, gastrointestinal effects. These adverse effects occur due to inhibition of the constitutive production of COX-2 in some tissues. Thus, in recent years, the safety of the use of NSAIDs in clinical practice has been questioned, due to the emergence of evidence that suggests a high risk of acute myocardial infarction, stroke, heart failure, renal failure, and arterial hypertension [30,38].

There is no absolute selectivity in COXs. Even a selective COX-2 inhibitor will also inhibit COX-1 when in high concentrations. Therefore, in all NSAIDs selective for COX-1 or COX-2, to different degrees, there is a risk of adverse cardiovascular side effects [30].

In September 2004, Merck removed Vioxx^®^ from the world market and in April 2005, the coxib study led the American Committee to conclude about the Cardiovascular Risk and suspension of Pfizer’s Bextra^®^ (valdecoxib). Celecoxib is marketed only with a black stripe indication and the adverse cardiovascular effects are explained in the package leaflet [36].

A derivative of celecoxib based a benzo[b]furan moiety was reported to demonstrate selective activity against COX-2. Besides, new molecules containing rhodanine and benzofuran scaffolds were designed, synthesized, and reported to exhibit dual COX-2, and 5-LOX inhibitory potential [39,40]. A recent patent survey reported a review focused on benzofuran inhibitors [41,42].

Since a large proportion of NSAIDs available on the market have significant undesirable effects, the need for new anti-inflammatory drugs contributes to the advancement of research for newer, safer, effective molecules with fewer side effects and from vegetal sources. Therefore, it can be observed that a significant number of substances of vegetal origin form part of the therapeutic arsenal of modern medicine. It is important to emphasize that due to the existence of a large number of species available for research, the success of the development of new naturally occurring anti-inflammatory drugs depends fundamentally on a multidisciplinary effort in the discovery of new leading molecules.

## 3. Plant Use and the Development of Drugs

The World Health Organization (WHO) estimates that approximately 65% of the world’s population incorporates traditional medicine (ethnobotanical uses) into medical care. Ethnobotanical studies over the years have allowed the association of highly diversified plants with biological activities, from observation, description, and experimental research, which has greatly contributed to the discovery of natural products with biological action. The use of medicinal plant-based natural compounds to treat many illnesses has become a great trend in clinical research. Polyphenolic compounds have drawn significant attention due to their modulation effects on inflammasomes [43]. These multi-protein complexes are associated with the initiation and progression of metabolic disorders and chronic diseases, such as cancer and neurodegenerative diseases [44].

Thus, plants have become the first source of substances for the development of new drugs, and a considerable part of the drugs prescribed in the world are derived from them [9,45,46,47]. Plants contain reservoirs of potential secondary metabolites that are the major sources of drugs, which intensifies the interest of transnational industries in the search for substances obtained from plant sources, particularly since the great majority of species have not been studied chemically or biologically [48].

The use of plants or plant products for medicinal purposes is mostly documented in books and, lately, on an enormous number of websites (the reliability of some of which must be examined carefully). In recent decades, hundreds of research and review articles have been published regarding the anti-inflammatory activities of plants (Table 2) [7,49,50,51,52].

Other plant species with anti-inflammatory properties have already been described in the literature. However, the parts of the plants used and the compounds responsible for the anti-inflammatory activity have not yet been fully elucidated.

Phytochemical studies carried out with the species *Myracroduo nurundeuva* Allemão, *Schinus terebinthifolius* Raddi, *Spondias mombin* L., *Spondias purpurea* L. and *Spondias tuberosa* Arruda, belonging to the Anacardiaceae family, detected the presence of several secondary metabolites. The most abundant are phenols, triterpenes, flavonoids, and cinnamic acid, which are responsible for their anti-inflammatory action [53,54,55].

The plants that make up the Euphorbiaceae family, such as the species *Euphorbiaceae acalypha* hispida Burm. f., *Acalypha indica* L., *Phyllanthus niruri* L., are rich mainly in phenolic compounds, saponins, tannins, and triterpenes, which are responsible for their anti-inflammatory action [56,57,58].

Research with the species *Ruellia asperula* (Mart. Ex Ness) Lindau (family Acanthaceae), Achyranthes *aspera* L., *Alternanthera brasiliana* (L.) Kuntze (family Amaranthaceae), *Himatanthus drasticus (Mart.) Plumel* (family Apocynaceae), *Matricaria chamomilla* L. (family Asteraceae), *Heliotropium indicum* L. (family Boraginaceae), *Momordica charantia* L. (family Cucurbitaceae), *Mimosa tenuiflora* (Willd.) Poir (family Leguminosae), *Borreria verticillata (L.) G.Mey*. (family Rubiaceae), *Solanum paniculatum* L. (family Solanaceae), and *Zingiber officinale Roscoe* (family Zingiberaceae) also indicates the existence of compounds with anti-inflammatory activity [59,60,61,62,63].

It is important to note that the extraction of plant materials is the first major step to test biological activities, presenting many advantages and some disadvantages compared to the isolation of pure active compounds [50]. When an entire extract is used, there is a good chance of synergism between active components that can be lost when each of these components is isolated. This synergism was discovered in several medicinal tests, including those for anti-inflammatory activity. On the contrary, the mixture of different compounds together may also lead to inhibitory effects, namely, that one component may reduce the biological activity of the other. In line with this assumption, some studies have shown that the anti-inflammatory activity of pure compounds (such as amentoflavone, pseudohypericin, and hyperforin) is higher than that of the extracts [50,64,65].

Medicinal plants are used instead of Non-steroidal anti-inflammatory drugs (NSAIDs) as the use of non-steroidal anti-inflammatory drugs is associated with several side effects, among which are unwanted effects on the gastrointestinal tract and the renal system. The biggest disadvantage of recently available potent synthetic drugs is concerning their toxicity and the reappearance of symptoms after discontinuation. Therefore, the screening and development of drugs with anti-inflammatory activity are necessary and there are many efforts to find anti-inflammatory drugs from medicinal plants [7].

Inflammation is a huge challenge for human kind. Although many anti-inflammatory drugs are available, it is believed that these drugs, such as opioids and analgesia inducing drugs like NSAIDs, are not useful in all cases and these drugs also produce side effects, so to overcome these problems, new drug molecules need to be discovered from plants. Plants have many phytoconstituents helpful in reducing inflammation and fewer side effects [7].

The objectives of the use of plants as therapeutic agents are: to concentrate and/or isolate bioactive substances for direct use as drugs; to produce bioactive compounds of novel or already known structures for semi synthesis to produce patentable entities of higher activity and/or lower toxicity; to use agents as pharmacological tools; and to use the whole plant or part of it as a herbal remedy [66].

It is worth mentioning that for the acquisition of new drugs, molecular diversity and biological function distinguish products of natural origin from synthetic products. The molecular diversity of natural products is far superior to that derived from synthesis processes, which, despite technological advances, are still restricted. This fact makes it possible for the chemical compounds present in plants to become potential drugs for different diseases [19].

An example of a phytotherapeutic anti-inflammatory agent is Acheflan^®^, indicated for the local treatment of inflammatory processes, and Daflon 500 mg^®^, a drug composed of a purified flavonoid fraction that presents venotonic and vasoprotective action [37]. Therefore, the study of the immunopharmacological activities of plant species has provided evidence on different extracts/fractions and chemical classes with high therapeutic potential, which represents a promising alternative to the inflammatory processes and diseases related to them, as well as a form of validation of their ethnobotanical use. Besides, data from the scientific literature have shown that molecules of plant origin present important anti-inflammatory activities and that many of their actions are related to the ability to inhibit the synthesis or action of cytokines, chemokines, and adhesion molecules, and arachidonic acid and nitric oxide pathways [67,68].

## 4. Secondary Metabolite Biosynthesis

Secondary metabolism is a set of reactions that have important biosynthetic intermediates, derived from biochemical processes that make up the primary glucose metabolism: such as glycolysis, the pentose pathway, the Krebs cycle, and photosynthesis. The main intermediates are shikimic acid, acetyl-coenzyme A (acetyl-CoA), and 1-deoxyxylulose 5-phosphate [69].

Shikimic acid is synthesized from a combination of phosphoenolpyruvate (via glycolysis) with 4-phosphate erythrosis (pathways pentoses or photosynthesis) and is a precursor to hydrolyzabletannins, coumarins, alkaloids derived from aromatic amino acids and phenylpropanoids, compounds that have the presence of an aromatic ring in common. These compounds are produced by the biochemical route called the shikimate pathway [70].

Acetyl-CoA is formed by oxidative decarboxylation of pyruvic acid (glycolysis) and its derivatives are fatty acids and polyketides; in turn, three acetyl-CoA molecules constitute mevalonic acid and 1-deoxyxylulose 5-phosphate originates from the combination of pyruvic acid and glyceraldehyde-3-phosphate (two intermediates of glycolysis); 1-deoxyxylulose 5-phosphate, together with mevalonic acid, are precursors to several terpenoids and steroids [71].

The secondary compounds produced from these intermediates are synthesized by biosynthetic routes called the acetate pathway, the mevalonate pathway, and the deoxyxylulose phosphate (DXP) pathway [72,73]. Through the acetate pathway, compounds called polyketides are formed (by the condensation of acetic acid units, giving rise to poly-β-keto chains). In this class are fatty acids, polyacetylenes, prostaglandins, macrolide antibiotics, and various aromatic compounds, such as anthraquinones and tetracyclines [74,75].

Other compounds such as some flavonoids and alkaloids are formed by a mixed route, that is, they have reaction intermediates from the acetate pathway and the shikimate pathway.

The mevalonate and deoxyxylulose phosphate pathways comprise the so-called terpenoids, steroids, triterpene and steroidal saponins, cardiotonic glycosides. Terpenoids are synthesized through the mevalonate pathway, in the cytoplasm [76].

Besides, the cycloartenol tetracyclic ring system is a precursor to many plant sterols. Modifications and substitutions that occur lead to the formation of a wide variety of compounds of natural origin, which present several important biological activities such as, for example, sterols, steroidal saponins, cardioactive glycosides, bile acids, corticosteroids, and mammalian sex hormones. The differences in biological activities presented by these compounds, which contain the tetracyclic skeleton in common, are attributed to the functional groups linked to the steroid nucleus, and to the stereochemical variations of that nucleus [77].

Saponins are glycosides of steroids or polycyclic terpenes. For aglycone, they are called steroidal saponins and triterpene saponins. Tripterpenic saponins correspond to a triterpenoid carbon skeleton pentacyclic, formed by the epoxidation and cyclization of squalene, in the conformation chair-chair-chair, and then carboxylations, oxidations, oxidation of methyl groups, the appearance of formyl (-CHO) or hydroxymethyl (-CH_2_OH) groups may occur [76].

The shikimate pathway is used only by microorganisms and plants and is a pathway for the synthesis of aromatic compounds, particularly aromatic amino acids: L-phenylalanine, L-tyrosine, and L-tryptophan. This pathway starts with phosphoenolpyruvate (from glycolysis) and D-erythrose phosphate (from the pentose pathway or photosynthesis), forming the shikimic acid and this, in turn, gives rise to aromatic compounds. The amino acids phenylalanine and tyrosine form phenylpropane, which is a basic unit present in many natural products (cinnamic acid, phenylpropanoids, coumarins, lignans, and flavonoids) and, together with tryptophan, are precursors to a wide range of alkaloid structures [74].

Phenylpropanoids are also derived from the route that begins with the formation of shikimic acid, giving rise to phenylalanine and tyrosine which, in turn, by the action of the enzyme phenylalanine ammonialyase, lose an ammonia molecule, resulting in the formation of cinnamic and 4-cumáric acids, respectively, and, through various reactions of reduction, oxidation, and cyclization give rise to several phenylpropanoids. Coumarins are derived from the metabolism of phenylalanine, one of its first precursors being 2-coumaric acid (orthohydroxy-cinnamic), which then undergoes lactonization of the side chain, giving rise to coumarin. Xanthones are heterocyclic phenolic compounds, which are based on the dibenzo-γ-pyrone nucleus. The biosynthesis of xanthones in higher plants is well determined, following the acetate-shikimate pathway. From two precursors, the formation of a common intermediate, a benzophenone, occurs with an aromatic ring originating from the shikimic acid route (B), which can characteristically contain hydroxyl groups in C5 and/or C7, and another from the malonyl-CoA route (THE) [78].

Chromones are heterocyclic compounds, which consist of the fusion of a benzene ring with a pyrone ring. The simplest compound in this family is the chromone itself (4*H*-chromen-4-one, 4*H*-1-benzopyran-4-one). The most abundant natural chromones are those that have substituents in the C2 and C3, by another benzene ring, being called flavones and isoflavones, respectively, and for this reason, their inclusion in the group of flavonoids is recurrent. These compounds constitute an important set of biologically active substances, mostly of natural origin, being present in great abundance in the plant kingdom [79].

Flavonoids are a class of polyphenolic compounds that differ from each other according to their chemical structure and particular characteristics. The biosynthesis of these compounds occurs through a via mist, between the shikimic acid and acetate pathways. Shikimic acid is the precursor to the starting compound for the synthesis of flavonoids, phenylalanine. After being deaminated by phenylalanine amonialiasis, this aromatic amino acid produces cinnamic acid, which by the action of 4-hydroxylase cinnamate is converted into p-coumaric acid (4-cumharic) [75].

Thus, in flavonoids, ring A is formed via acetate, while B results from the shikimate route and the three carbon atoms, which link ring A to B, derived from phenylpyruvate. Flavonoids can occur as aglycone or glycosides (a glucose molecule attached to the aromatic ring). The compounds belonging to this class are subdivided into subclasses: chalcones, dihydrochalcones, aurone, flavones, flavonols, dihydroflavonoids, flavanones, flavanol, flavandiol, anthocyanidins, isoflavonoids, bioflavonoids, and proanthocyanins. Chalcones are precursors to many derivatives of flavonoids, found throughout the plant kingdom, such as flavonones, and these, in turn, can then give rise to many other flavonoids, such as, for example, flavones, flavonols, anthocyanidins, and catechins. Other compounds can be formed by changes in hydroxylation, methylation, glycosylation, and dimethyl allylation in the two aromatic rings [75].

Anthraquinones are also called anthracenedione, anthracene derivatives, or hydroxyanthracene derivatives. The biosynthesis of these compounds has shikimic acid and acetate, or just acetate as precursors, and in the first case, shikimic acid reacts with α-ketoglutaric acid, resulting from the deamination of acid glutamic or the citric acid cycle, producing o-succinylbenzoic acid, 1,4-dihydroxy-2-naphthoic acid combines with mevalonic acid, giving rise to anthraquinone [74].

Tannins are a class of phenolic compounds of great complexity and importance. They are classified, according to their chemical structure, into two groups: hydrolyzable and condensed [77]. Gallic acid is a benzoic acid, derived from cinnamic acid, derived from the shikimate pathway. Gallic acid reacts with ureadin diphosphate-glucose (UDP-glucose), forming the intermediate β-glycocalin, which gives rise to 1,2,3,4,6-pentagaloyl-d-glucose, a molecule that, after oxidative transformations, leads to the formation of galotanins and ellagitannins [74].

There are three routes of biosynthesis of hydrolyzabletannins: in the first route, the addition of galloyl groups to 1,2,3,4,6-pentagaloyl-d-glucose is used, through *m*-depisidic bonds. In the second route, two neighboring gallic acid molecules (1,2,3,4,6-pentagaloyl-d-glucose) condense, and then this compound (monomer) can bind to others, forming dimers, trimers, and oligomers. Condensed tannins or proanthocyanidins are polymers of flavan-3-ol and/or flavan-3,4-diol (leukocyanidins), products of shikimate metabolism and acetate pathway (as seen in flavonoids). Dihydroflavonols are reduced to leucoanthocyanidins, which in turn give rise to catechins, which suffer a reduction in C4. Proanthocyanidins (condensed tannins) are probably caused by a condensation reaction between leucoanthocyanidins and catechins [74].

The biosynthesis of alkaloids always includes at least one amino acid, to these amino acids are also incorporated into other units from pyruvate, malonate, or mevalonate [80].

Thus, with such distinct precursors of biosynthetic origin, it is easy to understand the complexity and structural diversity of this class of metabolites, with more than 5000 alkaloids known today. Alkaloids are classified according to the amino acid that provides both the nitrogen atom and the fundamental part of the molecule’s skeleton. The aromatic amino acids histidine, tryptophan, tyrosine, and phenylalanine give rise to alkaloids with indole, isoquinolinic nuclei and anthranilic acid gives rise to quinolinic, quinazolinic, benzoxazinic alkaloids [74].

## 5. Anti-Inflammatory Molecules of Medicinal Plants and Mechanism of Action

**Figure 1:**
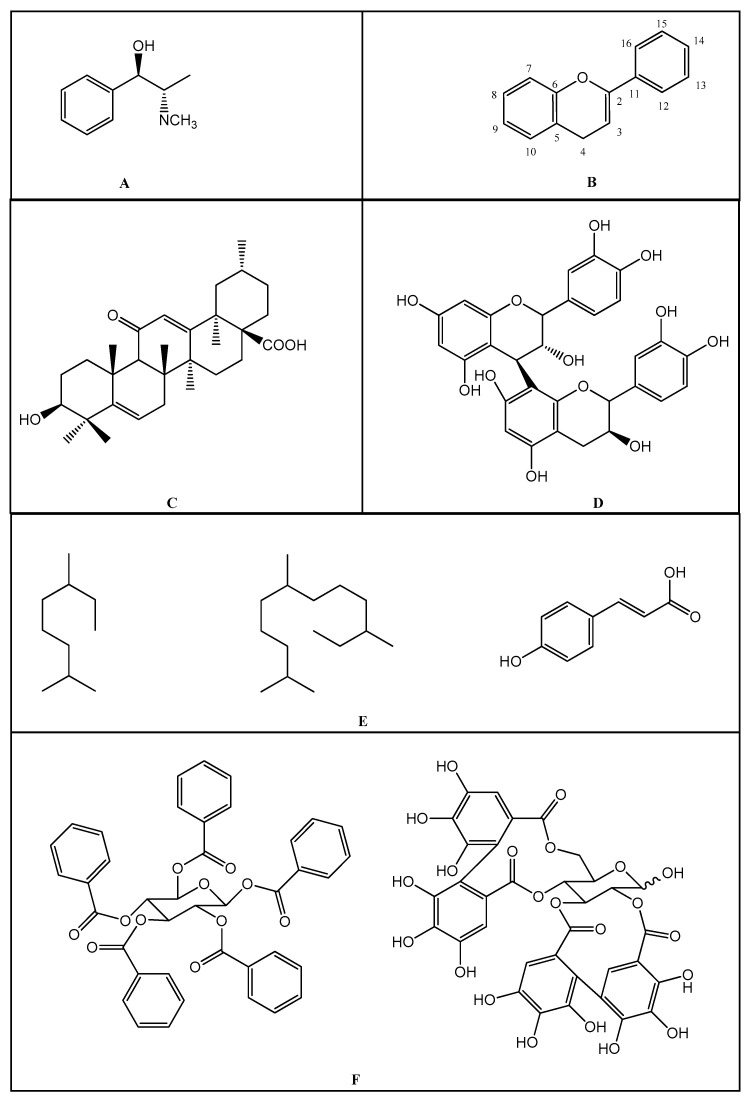
(**A**): Chemical structure of alkaloids: Ephedrine. (**B**): Basic Flavonoid Structure, (**C**): Chemical structure of saponins: Glycyrrhizin, (**D**): Structure of condensed tannin, (**E**): Structure of terpenoids and phenylpropanoids, (**F**): Structure of hydrolysable tannins: Galotannins and Ellagitannins [74,90].

Substances of plant origin, belonging to the most diverse chemical classes, have already demonstrated proven anti-inflammatory activity [9]. Among them, alkaloids, terpenes [81,82,83,84], and phenolic compounds such as tannins, lignans, coumarins, saponins, and especially the flavonoids stand out [83,84,85,86,87,88,89] (Figure 1).

The flavonoids represent a group of vegetal pigments with extensive distribution in nature, being available in fruits, seeds, flowers, and barks [91,92]. Their basic structure is the flavan nucleus, which consists of 15 carbon atoms arranged in 3 rings (C6-C3-C6) which are labeled as A, B, and C. Figure 1 presents the structure of the flavonoids and their numbering to distinguish the position of the carbons around the molecule [74].

The structural variation in ring C subdivides flavonoids into six main subclasses: flavonols (e.g., quercetin, kaempferol, myricetin), flavones (e.g., luteolin, apigenin), flavanols (e.g., catechin), flavanones (e.g., hesperetin), anthocyanidins (e.g., cyanidin, pelargonidin), and isoflavones (e.g., genistein, daidzein) [93].

Flavonoids have an anti-inflammatory capacity since they inhibit the production of inflammatory mediators by modulating the arachidonic acid pathway, inhibiting several enzymes such as ATPase, prostaglandin, cyclooxygenase, lipoxygenase, NADH oxidase, protein kinase, hydrolases, peroxidases, metallopeptidases, tyrosinases, and phospholipases. Thus, flavonoids have been the target of increasing interest as a potential therapeutic drug in inhibiting or even decreasing inflammatory activity [88,91].

Although an expressive number of flavonoids are present in plants in the form of glycosides, consignments about the biological activities of flavonoids have been made mainly for aglycones. Some research on the structure-activity relationship and the inhibition of inflammatory mediators can also be found in the scientific literature. It has been reported that the presence of hydroxylations at the 3′ and 4′ positions of the B ring of the flavonoid structure enhances the modulatory action on the inhibitory activity of TNF-α [94], while the presence of only one hydroxyl minimizes inhibition and the absence of this group overrides the inhibitory effect. To inhibit iNOS or nitric oxide (NO) production, the presence of at least three hydroxyls is required [95]. This is the case of luteolin and its derivatives that have a catecholic pattern of oxygenation in ring B and also oxygenations in ring A. Oxygenation at position 5 of ring A contributes significantly to anti-inflammatory activity [96].

The anti-inflammatory potential of 9 flavonoid aglycones was evaluated: kaempferol, quercetin, apigenin, chrysin, diosmetin, luteolin, daidzein, genistein, and hesperetin. Among them, luteolin was the most active in the inhibition of NO and TNF-α [95].

Quercetin is found in high concentrations in plants such as *Allium cepa, Camellia sinensis, Hypericum perforatu,* and *Podophyllum peltatum*. This compound acts by promoting a significant reduction in edema volume in both acute and chronic models; with effects comparable to those of phenylbutazone [50].

The flavonoids rutin, quercetin, and hesperidin were found to have anti-inflammatory effects. Plants such as onions (*Allium cepa*) contain a high concentration of quercetin and studies confirmed the anti-inflammatory activities of onion juice and extracts. *Abutilon indicum* also contains high amounts of quercetin and has significant anti-inflammatory activity. Besides, garlic contains large amounts of allicin which exerts potent anti-inflammatory effects [97,98,99].

Investigations involving kaempferol, quercetin, and aromadendrene glycosides and their anti-inflammatory activity due to suppression of NO levels in LPS-stimulated microglial cells indicated that glycosylation attenuated the aglycone suppressor activity. Although attenuated, some of the glycosides, depending on the position and degree of glycosylation, retained the inhibitory capacity of NO production, which suggests that the glycosylation of flavonoids should be considered as an important modulator of biological activity [100].

Although other authors have shown that the glycosidic portion of biologically active substances may be a crucial factor for their biological activity, in many situations, glycosylation only improves pharmacokinetic parameters [101]. The presence of methoxyl as substituents in flavonoids may considerably interfere with their anti-inflammatory activity; 41 synthetic chalcones were studied, verifying that the most active structures have a methoxyl in the position adjacent to a carbonyl [102].

Another group of compounds known for their anti-inflammatory activity is alkaloids. They usually contain nitrogen in a heterocyclic ring, in a state of negative oxidation, with an amine group that gives them a basic character [103]. The alkaloids are classified according to the nature of the nitrogen atom present in their structure. Alkaloids that have a nitrogen atom in a heterocyclic ring are called true alkaloids. Substances with nitrogen atoms not belonging to a heterocyclic ring are called protoalkaloids and substances with and without heterocyclic rings that are not derived from amino acids are called pseudoalkaloids [82].

Alkaloids can also be classified according to their biogenetic precursors, which confer their structural characteristics, such as indole alkaloids. Alkaloids are derived from amino acids such as ornithine, lysine, tyrosine, and tryptophan. Ornithine is a precursor for pyrrolidine and tropic alkaloids, while lysine gives the piperidine alkaloids. Tyrosine and tryptophan are formed by the shikimic acid pathway and originate the isoquinolinic and indole alkaloids respectively [104]. Alkaloids are considered the most toxic among the several active principles. Some are known and used in scientific therapy, such as morphine, ergotamine, and ephedrine [105].

Saponins, such as glycyrrhizin, on the other hand, are steroid glycosides or polycyclic terpene used for the synthesis of cortisone (anti-inflammatory) and sex hormones. They increase the uptake and use of certain minerals [106] and can form complexes with steroids, proteins, and membrane phospholipids determining important biological properties such as changes in cell membrane permeability [77].

There is also the class of tannins. Plants that have a high tannin content are used in traditional medicine for the treatment of various diseases such as diarrhea, high blood pressure, rheumatism, wounds, kidney, and urinary problems, and inflammatory processes. The pharmacological activities of tannins are due to their high complexity with metallic ions (iron, manganese, copper, and others), antioxidant activity, and the ability to complex with other molecules such as proteins and polysaccharides [107].

The tannins are classified into two main groups, whose structures are very different from each other, although all of them have in the molecule polyhydroxyphenols or their derivatives. Those belonging to the first group are called hydrolysable tannins, which include galotannins and ellagitannins, polymers derived from gallic and ellagic acids [108].

The constituents of the second group are called condensed tannins. They are found in greater proportions and importance in food. At higher concentrations, they give fruits and other food astringent characteristics [109].

There are also terpenes, which exhibit pharmacological properties, such as anti-inflammatory and antinociceptive, inhibit platelet aggregation, and interfere at an intracellular level in several steps of the transduction mechanism. The chemical components of volatile oils can be divided into two groups: terpenoid derivatives (representing the union of two, three, or more isoprene units) formed by the mevalonic acid-acetate route, and the phenylpropanoid derivatives, aromatic compounds formed by the shikimic acid pathway [110].

Triterpenes such as α/β-amyrin acetate, nimbin, filicene, and oleanolic acids are found in high concentrations in plants such as *Thymus serpyllum, Syzygium aromaticum, Salvia triloba, Rosmarinus officinalis, Origanum majorana, Ligustrum lucidum, Lavandula latifolia*. These compounds act by promoting a significant reduction in the volume of edema; effects comparable to those of hydrocortisone. Besides, several terpenes and polyphenols have been identified in high concentrations in *Nepenthes mirabilis* (Lour.) Rafarin (a carnivorous plant), promoting a significant reduction in the levels of IL-6, IL-12, and TNF-α [50].

Several pathologies, such as the inflammatory process, can be aggravated by the formation of free radicals and generate tissue lesions by promoting oxidation [111]. It is well-known that oxidative stress plays important role in endothelial dysfunction, lung disease, gastrointestinal dysfunction, and atherosclerosis, and inflammatory symptoms are involved in all these disorders [112,113]. Excessive pro-inflammatory cytokines and mitochondrial dysfunction induce oxidative stress, characterized by an imbalance between the effectiveness of antioxidant defense and the speed of ROS generation, causing a net overload of oxidants [114,115,116].

Antioxidant compounds may reduce oxidative stress, minimizing the incidence of these pathologies. The search for new antioxidant agents from plant sources used in the human diet and folk medicine against inflammation and infection may lead to the discovery of natural molecules with high anti-inflammatory potential in vitro and in vivo. These substances could justify the popular use of these plant species with anti-inflammatory properties [117].

Free radicals are organic or inorganic molecules that have atoms that contain one or more unpaired electrons [118]. The majority of free radicals observed are superoxide (O_2_^−^), hydroxyl (OH^−^), hydroperoxide (HO_2_^−^), nitric oxide (NO^•^), and nitrogen dioxide (NO_2_^•^). Among these, the hydroxyl radical is the most reactive in the induction of cellular lesions and hydrogen peroxide can cross the nuclear membrane and induce damage in the DNA molecule [119,120].

Free radicals induced by peroxidation are implicated in the pathogenesis of chronic inflammation that can trigger various pathological conditions such as arthritis and cancer, among others [121]. It is important to mention that inflammation has been linked to an increased risk for the development of different types of tumors such as gastric mucosal lymphoma, colon cancer, gastric cancer, and prostate cancer. Many factors lead to chronic inflammation such as microbial infections, autoimmune diseases, inflammatory conditions, physical injuries, or chemical compounds, such as free radicals [122]. Besides, inflammatory processes also promote an increase in the concentration of free radicals in the body [123].

Lipid peroxidation is the process by which reactive oxygen species (ROS) attack the polyunsaturated fatty acids of the phospholipids of cell membranes, disintegrating them and allowing the entry of these species into intracellular structures. In this scenario, phospholipase that has been activated by toxic species disintegrates the phospholipids and this causes the release of unsaturated fatty acids. As in the formation of ROS, the processes of lipo peroxidation are not always harmful, since their products are significant in the cascade reaction from arachidonic acid and, therefore, in the inflammatory response. Research has shown that flavonoids inhibit lipid peroxidation, in vitro, in the initiation stage, due to its action as an antioxidant [124].

In this context, inflammation is clinically defined as a pathophysiological process characterized by redness, increased heat, swelling, pain, and loss of function. These signs occur as a response of the organism to the invasion of infectious agents or physical injury. Both the innate immune response and acquired are involved in the inflammatory process, generating numerous local and systemic effects. Depending on the time of evolution, it can be acute or chronic, although sometimes the conventional patterns cannot detect a previous event [125,126]. The innate immune system is the foremost defense mechanism against invading microorganisms and cancer cells, involving the activity of various cells including macrophages, mast cells, and dendritic cells. The adaptive immune systems involve the activity of more specialized cells such as B and T cells which are responsible for eradicating invading pathogens and cancer cells by producing specific receptors and antibodies [50].

During the inflammatory process, vascular and chemical events are identified [127,128]. Vascular events occur in the microcirculation, causing several modifications such as elevation of blood flow and capillary permeability, among others. These alterations are local effects of chemical mediators, which are plasma enzyme systems, cytokines, mast cell products, platelets, leukocytes, and products of arachidonic acid metabolism [128,129]. Thus, the inflammation events that underlie these manifestations are induced and regulated by a large number of chemical mediators, including kinins, eicosanoids, complement proteins, histamine, and monokines [7].

During the inflammatory process, short-term and rapid-onset reactions lasting 1 to 2 weeks, belonging to the group of acute inflammation, may be recognized. On the other hand, reactions that persist for longer than months or years and that have a slow and insidious speed, belong to the group of chronic inflammation [130]. Both can be restricted to local phenomena or involve systemic phenomena. The characteristics, extent, and severity of the inflammatory process depend on factors related to the host (nutritional status and genetic factors) and the nature and pathogenicity of the aggressor agent [92].

Flavonoids also act by modulating the induced nitric oxide synthase enzyme and the cells involved with inflammation, inhibiting the production of proinflammatory cytokines and modulating the activity of arachidonic acid pathways, such as cyclooxygenase (COX), lipoxygenases (LOX), and phospholipase A2 [131].

Phospholipases A2 (PLA2) formsone of the main groups of mediators of the inflammatory process, through the production of arachidonic acid. By inhibiting the action of phospholipase, the onset of the inflammatory process is inhibited. Besides, free arachidonic acid can be metabolized to various lipid mediators known as eicosanoids via the lipoxygenases and cyclooxygenase pathways [132,133]. Furthermore, arachidonic acid can be metabolized to prostaglandins and epoxyeicosatrienoic acids by cyclooxygenase-2 (COX-2) and cytochrome P450 (CYP), respectively [114,134,135].

Thus, arachidonic acid metabolites play a vital role in inflammation. In inflammatory conditions, arachidonic acid is released from membrane phospholipids by the enzyme phospholipase A2 and metabolized by cyclooxygenases, lipoxygenases, and cytochrome P450s (CYPs) to prostaglandins/thromboxane, leukotrienes, and epoxy/hydroxy-metabolites such as epoxyeicosatrienoic acids and other EpFAs, respectively [114,136,137]. Cyclooxygenase (COX), an enzyme responsible for the generation of prostaglandins (PG) from arachidonic acid, which is released from phospholipids of the cell membrane by phospholipase, has an induced form (COX-2) and a constitutive form (COX-1) [114].

COX-1 is essential for maintaining the normal physiological state of many tissues, including the protection of the gastrointestinal mucosa; control of renal blood flow; homeostasis; autoimmune responses; pulmonary and central nervous system functions; and cardiovascular and reproductive diseases [30]. COX-1 stimulus regulates the normal physiological processes, being responsible for the synthesis of prostaglandins.

In contrast, COX-2 is not detectable in tissues under normal physiological conditions, but its expression increases considerably during inflammation or mitogenic stimulation [138]. COX-2, induced in inflammation by various stimuli—such as cytokines, endotoxins, and growth, gives rise to prostaglandins, which contribute to the development of edema, flushing, fever, and hyperalgesia [30]. Thus, COX-2 is inducible or regulated by inflammatory stimuli, such as interleukin-1b, tumor necrosis factor, and lipopolysaccharide. This enzyme is associated with the production of prostaglandin E2 and prostacyclin that evoke peripheral and systemic inflammatory symptoms [32,33,34,35].

Therefore, the activation of these enzymes stimulates intracellular signals (NFkB, p38 or MAPKs, which modify the expression of pro-inflammatory cytokines, for example, interleukin 1 beta (IL1b), interleukin 6 (IL6), and tumor necrosis factor-alpha (TNF-α) adhesion proteins and chemokines, leading to stimulation and activation of immune cells [122]. Thus, the metabolites of COX-2 are involved in inflammatory disorders. Studies indicate that inhibition of COX-2 or soluble epoxidehydrolase is beneficial for inflammation [114,139,140,141,142,143,144,145,146,147].

In 2002, a new isoform was discovered: COX-3 [38,138,148]. Therefore, many researchers focus on the development of new and selective inhibitors of pro-inflammatory enzymes and cytokines [149]. However, it remains poorly characterized [30].

Inhibition of COX has been considered an important target of possible drugs for the treatment of inflammation [35]. Inhibition of COX by agents such as acetylsalicylic acid seems to be responsible for the lack of balance of arachidonic acid metabolites, raising the products derived from lipoxygenase, leukotrienes, with bronchoconstriction and proinflammatory properties [150].

Thus, it is important to note that the successful development of new naturally occurring anti-inflammatory drugs depends mainly on a multidisciplinary effort to find new molecules due to the existence of a large number of species available for research.

## Figures and Tables

**Table 1:** Classification of NSAIDs [29].

Salicylates	Indoleacetic Acid Derivatives	Aryl Acetic Derivatives	Enolic Acids	
Acetylsalicylic acid Lysine clonixinate Benorilate Diflunisal Salicylamide Etersalate Salsalate or salicylic acid	Acemethacin Glucamethacin Indomethacin Proglumethacin Oxamethacin Sulindac Tolmetin Difenpiramide	Aceclofenac Diclofenac Etodolac Fentiazac Ketorolac Bufexamac Lonazolac Alclofenac Zomepirac	**Oxicans:** Droxicam Meloxicam Piroxicam Tenoxicam Oxaprozin Lornoxicam	**Pyrazolones:** Phenylbutazone Mofebutazone Oxyphenbutazone Kebuzone Metamizole (Dipyrone) Feprazone Nifenazone Suxibuzone Aminophenazone
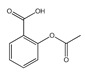	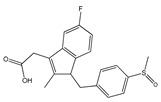	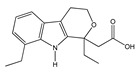	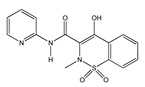	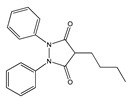
Aspirin	Sulindac	Etodolac	Piroxicam	Phenylbutazone
**Arylpropionic Derivatives**		**Phenemates**	**Others**	
Butibufen Phenoprofen Phenobufen Flurbiprofen Benoxaprofen Suprofen Ibuprofen Ibuproxam	Ketoprofen Dexetoprofen Pyprophene Indoprofen Naproxen Oxaprozin Tiaprofen Dexibuprofen Phenoprofen Flunoxaprofen Alminoprofen	Meclofenamic acid Mefenamic acid Flufenamic acid Tolipanic acidNiflumic acid Etofenamate	Nabumetone Glucosamine Diacerhein Nimesulide Proquazone Azapropazone Benzidamine Orgotein Feprazone Morniflumato Tenidap Glucosaminoglycan	**Coxibs:** Celecoxib Rofecoxib Parecoxib Valdecoxib Etoricoxib 4-Aminophenol Paracetamol (Acetaminophen)
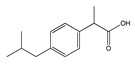	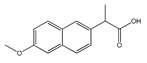	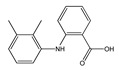	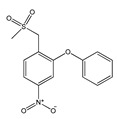	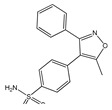
(*S*)-Ibuprofen	Naproxen	Mefenamic acid	Nimesulide	Valdecoxib

**Table 2:** Anti-inflammatory activity of some medicinal plants.

Number	Botanical Name	Plant/Family	Parts Used	Constituent Compounds
01	*Acacia catechu*	Mimosaceae	Bark, wood, flowering tops, gum.	Tannin, gum, catechuic acid
02	*Azadirachta indica*	Meliaceae	Leaf, root, oil, seed, gum, fruit, flower.	Margosine, bitter oil, azadirachtin.
03	*Caesalpinia crista*	Caesalpiniaceae	Seeds, root, leaf, root bark.	Oleic, linoleic, palmitic, stearic acid, phytosterols.
04	*Cassia angustifolia*	Caeasalpinaceae	Pods, dried leaves.	Emodin, eatharitin, mucilage, senna-picrin, opleanic acid.
05	*Coriandrum sativum*	Umbelliferaeapiaceae	Leaf, bark, flower	Tannin, cathartin, malic acid, cathartin, albuminoids.
06	*Cuscuta reflexa*	Convolvulaceae	Plant, seed, fruit, stem.	Cuscutine, flavonoid, glucoside, bergenin, coumarin.
07	*Enicostema littorale*	Gentianaceae	Whole plant.	Alkaloids, gentiocrucine
08	*Erythrina variegate*	Papilionaceae	Leaves, bark, roots, flower.	2-Hydroxygenistein, genistein.
09	*Euphorbia hirta*	Euphorbiaceae	Plant, roots, leaves	Ascorbic acid, β-amyrin, choline, inositol, linoleic acid, β-sitosterol.
10	*Euphorbia tirucalli*	Euphorbiaceae	Root, plant (milk, juice).	β-sitosterol, ellagic acid, citric acid, malic acid, eupholglucose.
11	*Fagonia cretica*	Zygophyllaceae	Leaves, twigs, bark.	Betulin
12	*Ficus benghalensis*	Moraceae	Aerial roots, bark, seeds, leaves, buds, fruits, latex.	Skin, fruits contain 10% tannin.
13	*Ficus carica*	Moraceae	Fruit, root.	Alkaloids, ascorbic acid, caffeic acid, niacin, linoleic acid, lutein, β-carotene, pantothenic acid, β-amyrin.
14	*Ficus religiosa*	Moraceae	Bark, leaves, fruits, tender shoots, seeds.	The bark contains tannins, rubber, wax.
15	*Foeniculum vulgare*	Apiaceae	Fruit, root, seeds, leaves.	Ascorbic acid, estragole, coumaric acid, caffeic acid, α-terpinene, scoparone, scopoletin, cynarin, D-limonene, α-phellandrene.
16	*Gentiana kuroo*	Gentianaceae	Rhizomes (roots)	Gentiopicrine, gentianic acid
17	*Gloriosa superba*	Liliaceae	Rhizome, tuber, leaves, flower	Choline, colchicine, stigmasterol, salicylic acid, 2-methylcolchicine.
18	*Glycyrrhiza glabra*	Papilionaceae	Roots, leaves.	Genistein, eugenol, bergapten, glycyrrhizin, acetophenone, estragole, camphor, ascorbic acid, apigenin, anethole.
19	*Gmelina arbórea* Roxb	Verbenaceae	Whole plant.	Betulin
20	*Grewia asiatica*	Tiliaceae	Leaves, roots, fruits, bark.	Betulin
21	*Hibiscus rosa-Sinensis*	Malvaceae	Buds, roots, leaves, flower	Quercetin, ascorbic acid.
22	*Hygrophila auriculata*	Acanthaceae	Roots, leaves, seeds.	Oleic and linoleic acids in seed oil, palmitic acid, stearic acid.
23	*Manihot esculenta*	Euphorbiaceae	Tuberous roots.	Ascorbic acid, palmitic acid, lauric acid, stearic acid, oleic acid.
24	*Martynia annua*	Pedaliaceae	Fruits, leaves.	Pelargonidin-3,5-diglucoside, cyanidin-3-galactoside, semi-drying oil.
25	*Momordica charantia*	Cucurbitaceae	Whole plant	5-Hydroxytryptamine, alkaloids, ascorbic acid, β-carotene, cholesterol, lutein, diosgenin, lanosterol, lycopene, momordicin, charantin niacin, momordicoside.
26	*Moringa oleifera*	Moringaceae	Roots, bark, leaves, seeds.	Choline, moringinine, myristic, ascorbic acid, β-carotene, niacin, oleic acid, spirochin, stearic acid, tocopherol, vanillin.
27	*Nelumbo nucifera*	Nymphaeaceae	Whole plant.	Anonaine, ascorbic acid, β-carotene, copper, erucic acid, glutathione, hyperoside, myristic acid, nuciferine, oxoushinsunine, rutin, stearic acid, trigonelline, kaempferol, D-catechin.
28	*Nicotiana tobacum*	Solanaceae	Leaves.	1,8-Cineole, 4-vinylguaiacol, acetaldehyde, acetophenone, alkaloids, anabasine, nicotinic acid, nicotine, scopoletin, quercitrin, sorbitol, tocopherol stigmasterol, trigonelline.
29	*Nigella sativa*	Ranunculaceae	Seeds.	α-spinasterol, ascorbic acid, β-sitosterol, carvone, D-limonene, linoleic acid, myristic acid, methionine, nigellone, stearic acid, stigmasterol, tannin, thymoquinone, hederagenin.
30	*Ocimum basilicum*	Laminaceae	Whole plant	Acetic acid, ascorbic acid, aspartic acid, apigenin, arginine.
31	*Plumbago zeylanica*	Plumbaginaceae	Root, leaves, root, bark.	Plumbagin, droserone, 3-chloroplumbagin, chitranone, zeylinone, elliptione, isozeylinone.
32	*Portulaca oleraceae*	Portulaceae	Stem, leaves, seeds.	Oleracins I and II, acylated betacyanins, carbohydrate, galacturonic acid, mucilage.
33	*Pterocarpus marsupium*	Fabaceae	leaves, flower, gum Heartwood,	Alkaloids, gum, essential oil, semi-drying fixed oil.
34	*Solanum melongena*	Solanaceae	Roots, leaves, tender fruits.	Ascorbic acid, alanine, arginine, caffeic acid.
35	*Solanum nigrum*	Solanaceae	Whole plant.	Solenin, solasodine.
36	*Stereopermum suaveolens*	Bignoniaceae	Roots, flower	Mucilage, albumin, sugar, wax, lapachol, dehydrotectol, β-sitosterol, *n*-triacontanol.
37	*Tephrosia purpurea*	Fabaceae	Whole plant	Tephrosin, betulinic acid, lupeol, rutin.
38	*Terminalia chebula*	Combretaceae	Mature, immature fruits.	Ascorbic acid, gallic acid, ellagic acid, chebulic acid.
39	*Thespesia populnea*	Malvaceae	Whole plant	Gossypol, herbacetin, kaempferol.
40	*Thespesia populneoides*	Malvaceae	Whole plant	Populneol, gossypol, kaempferol, quercetin-5-glucoside, calycopterin, kaempferol-5-glucoside, kaempferol-3-gluoside.
41	*Tinospora cordifolia*	Menispemaceae	Stem	Alkaloids, starch.
42	*Vernonia cinerea*	Asteraceae	Whole plant	Linoleic acid, lupeol, vernolic acid.
